# Supplementary material for: Photoluminescence Modulation of Ruddlesden-Popper Perovskite via Phase Distribution Regulation
Source: Nanomaterials (Basel). 2023 Jan 31;13(3):571. doi: 10.3390/nano13030571 (PMC9921436; doi:10.3390/nano13030571)
Supplement: Supplementary file 1 [file nanomaterials-13-00571-s001.zip › nanomaterials-2167104-supplementary.pdf]

Supplementary Materials: Photoluminescence Modulation of Ruddlesden -Popper Perovskite via Phase Distribution Regulation

Xinwei Zhao, Ting Zheng*, Weiwei Zhao, Yuanfang Yu, Wenhui Wang and zhenhua Ni*

School of physics, Southeast University, Nanjing 211189,
Xinwei Zhao, Ting Zheng and Weiwei Zhao contributed to this work equally.
* Correspondence: zhni@seu.edu.cn and zhengtingdn@163.com

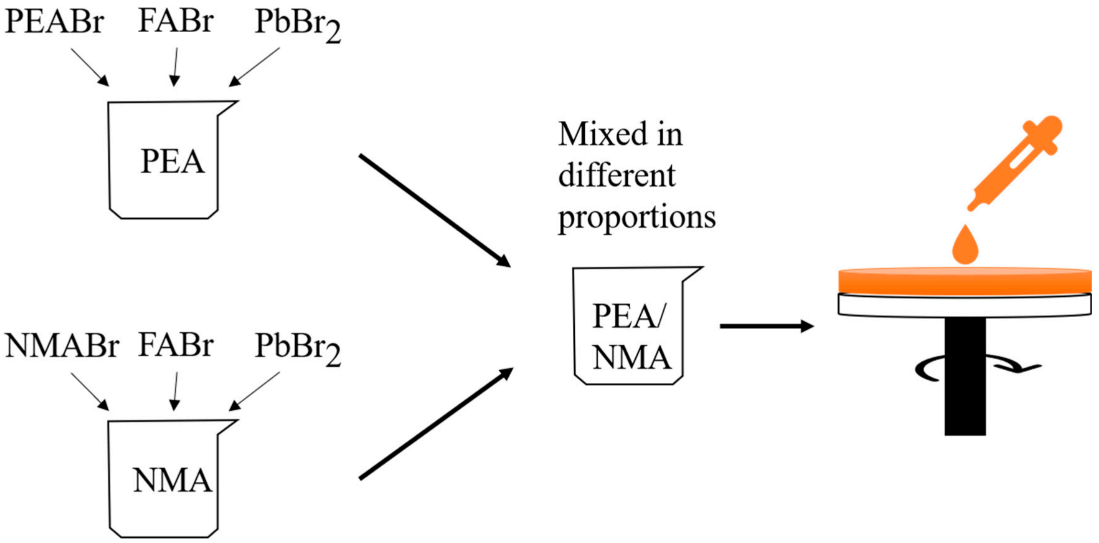

Figure S1. Schematic diagram of preparation process of PEA and NMA mixed perovskite film

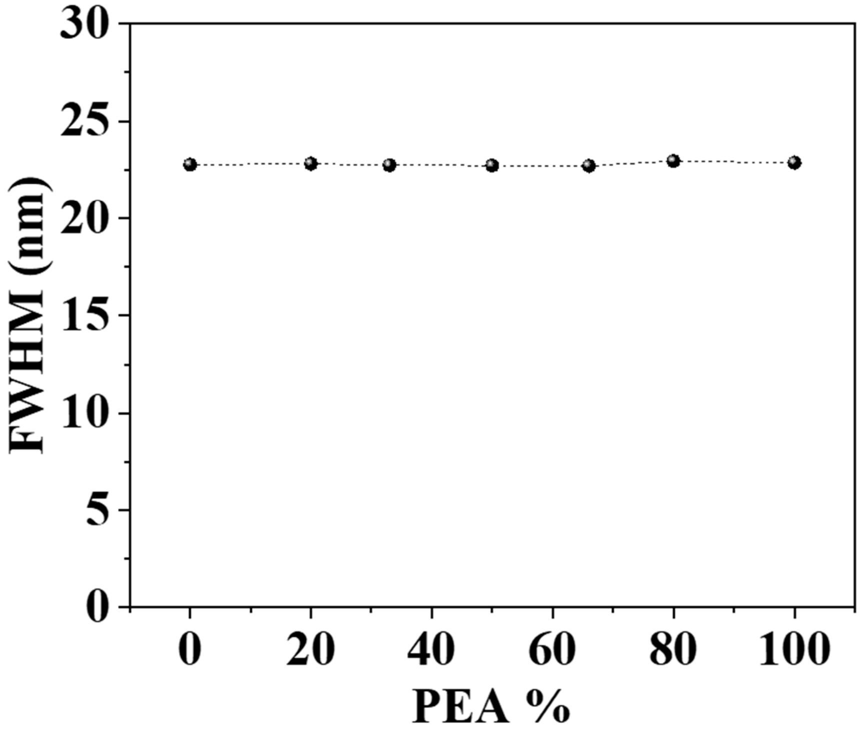

Figure S2. Full width at half maximum of PL as a function of PEA concentration**.**

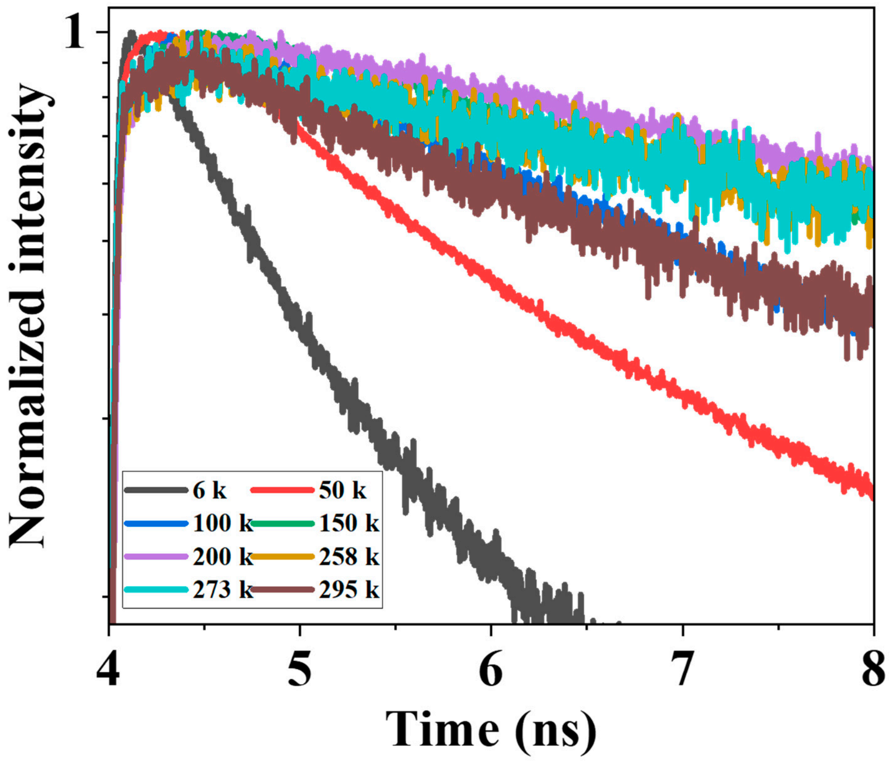

Figure S3. TRPL spectra of 0 % PEA sample with different temperature.

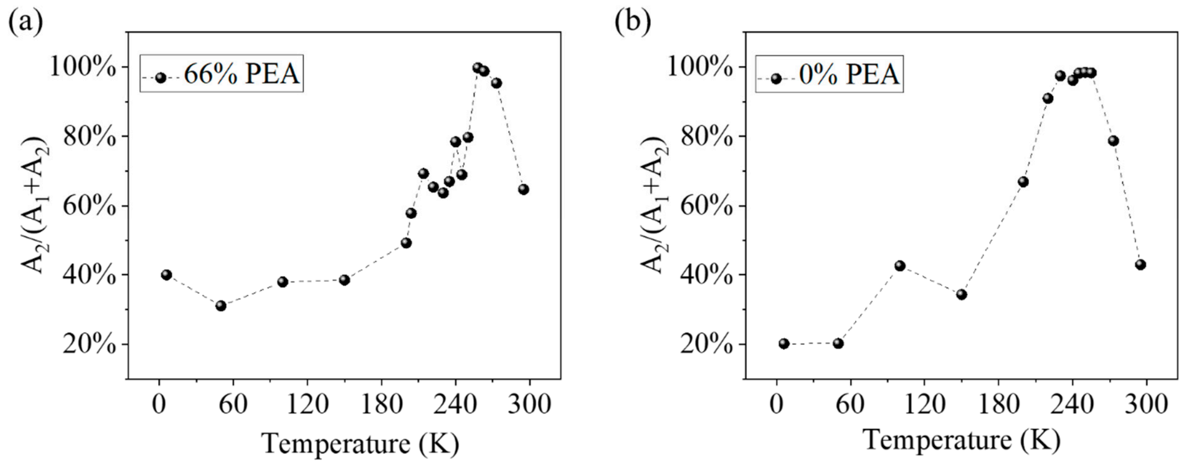

Figure S4. The weights of τ_2 as a function of temperature by fitting the data of 66 % PEA RPP in Figure 3c (a) and 0 % PEA RPP in Figure S1 (b).

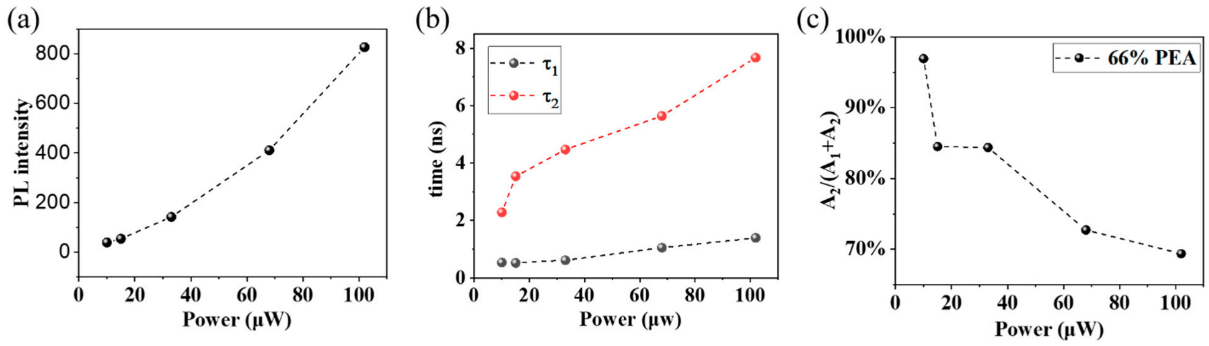

Figure S5. Power-dependence TRPL of 66% PEA RPP at ~ 250 K. (a) The PL intensity with various power; (b) The fitting results of τ_1 and τ_2 with various power. (c) The weight of τ_2 with various power.
